# KLF15 and PPAR*α* Cooperate to Regulate Cardiomyocyte Lipid Gene Expression and Oxidation

**DOI:** 10.1155/2015/201625

**Published:** 2015-02-26

**Authors:** Domenick A. Prosdocimo, Jenine E. John, Lilei Zhang, Elizabeth S. Efraim, Rongli Zhang, Xudong Liao, Mukesh K. Jain

**Affiliations:** ^1^Case Cardiovascular Research Institute and Harrington Heart & Vascular Institute, Cleveland, OH 44106, USA; ^2^Department of Medicine, University Hospitals Case Medical Center and Case Western Reserve University School of Medicine, Cleveland, OH 44106, USA

## Abstract

The metabolic myocardium is an omnivore and utilizes various carbon substrates to meet its energetic demand. While the adult heart preferentially consumes fatty acids (FAs) over carbohydrates, myocardial fuel plasticity is essential for organismal survival. This metabolic plasticity governing fuel utilization is under robust transcriptional control and studies over the past decade have illuminated members of the nuclear receptor family of factors (e.g., PPAR*α*) as important regulators of myocardial lipid metabolism. However, given the complexity of myocardial metabolism in health and disease, it is likely that other molecular pathways are likely operative and elucidation of such pathways may provide the foundation for novel therapeutic approaches. We previously demonstrated that Kruppel-like factor 15 (KLF15) is an independent regulator of cardiac lipid metabolism thus raising the possibility that KLF15 and PPAR*α* operate in a coordinated fashion to regulate myocardial gene expression requisite for lipid oxidation. In the current study, we show that KLF15 binds to, cooperates with, and is required for the induction of canonical PPAR*α*-mediated gene expression and lipid oxidation in cardiomyocytes. As such, this study establishes a molecular module involving KLF15 and PPAR*α* and provides fundamental insights into the molecular regulation of cardiac lipid metabolism.

## 1. Introduction

The adult heart is an endurance machine requiring large amounts of energy to meet its metabolic demand for sustained function [1]. A multitude of carbon substrates feed the mammalian myocardium to efficiently match fuel supply with energy demand, thus making the heart one of the largest consumers of energy in the body [1, 2]. Of these carbon substrates, oxidative catabolism of fatty acids (FAs) is the preferred fuel source in the healthy myocardium accounting for ~70% of the ATP generated in the mitochondria with the remainder coming from glucose, ketones, and lactate. Importantly, given the unrelenting demand for mechanical power, the myocardium is endowed with the ability to rapidly adjust its metabolism to substrate availability. As such, the metabolic myocardium has evolved robust molecular and allosteric mechanisms to adjust to various physiologic and pathologic milieus in order to meet its unrelenting need for energy [3]. For example, during periods of nutrient deprivation or increased energetic need (e.g., exercise) the heart augments lipid flux and utilization as a means to guard against energy exhaustion. In addition, under pathologic conditions such as insulin resistance/diabetes, cardiac uptake and oxidation of lipids are not appropriately balanced and glucose use is reduced [4]. Consequently, the diabetic heart experiences lipotoxicity and cellular stress that may contribute to a myopathic phenotype [5]. Finally, the importance of metabolic plasticity and impaired lipid utilization has been observed in human and experimental models of heart failure [2, 3, 6]. These observations thus underscore the importance of understating the molecular circuitry that governs cardiac metabolism to provide important insights into the fundamental mechanisms by which the heart utilizes fuel sources.

Cardiac lipid metabolism involves the coordination of sarcolemmal FA uptake, mitochondrial transport, and *β*-oxidation [1]. FA uptake is facilitated by cell surface transporters including fatty acid translocase (CD36) and fatty acid transport protein (Fatp). Cytosolic FA is then converted into a membrane impermeable long-chain acyl CoA moiety which acts as substrate for the outer mitochondrial membrane associated protein carnitine palmitoyltransferase-1 (CPT1) generating a membrane permeable long-chain acylcarnitine moiety. This acylcarnitine is translocated into the mitochondria matrix by the carnitine:acylcarnitine translocase (Slc25a20) where acylcarnitine is converted back to long-chain acyl CoA by CPT2, thus allowing for entry into the *β*-oxidation cycle with each spiral shortening the fatty acyl moiety by two carbons and producing acetyl CoA as well as reducing equivalents (FADH2, NADH) which feed into the tricarboxylic acid (TCA) cycle and electron transport chain (ETC), respectively.

Myocardial FA oxidation (FAO) is controlled at numerous enzymatic steps and is dependent on FA supply, demand, uptake, mitochondrial transport rate, and the rate of *β*-oxidation [1, 7]. In addition to posttranslational control of protein function, this metabolic machinery is also under robust transcriptional regulation that tightly couples gene expression with nutrient supply and energetic demand [2, 3, 6]. From a transcriptional standpoint, studies on control of cardiac metabolism have focused largely on nuclear receptors (NR) [8]. In particular, experimental work over the past several years has demonstrated key roles for the peroxisome proliferator-activated (PPAR) family of NRs, in particular PPAR*α*. PPAR*α* is ligand-activated and heterodimerizes with retinoid X receptor (RXR) that binds to PPAR response elements (PPRE) on target promoters to regulate gene expression [9]. PPAR*α* is highly expressed in tissues with high capacity for FAO including heart, skeletal muscle, liver, and brown adipose. Canonical PPAR*α* transcriptional targets in the myocardium include Cd36 and Fatp1 along with dehydrogenases for medium, long, and very long chain acyl-CoAs (Acadm, Acadl, and Acadvl) [9, 10]. The importance of PPAR*α* in regulating FAO in the heart has been demonstrated using both gain- and loss-of-function studies in mice [11–13]. Systemic deletion of PPAR*α* results in attenuated cardiac FAO rates and age-related cardiac fibrosis whereas mice with high levels of cardiac-specific PPAR*α* overexpression show augmented fatty acid uptake and oxidation, accumulation of intracellular triglycerides, and left ventricular hypertrophy. In sum, ligand activation of PPAR*α* is an essential pathway that regulates cardiac lipid utilization.

Kruppel-like factors (KLFs) are members of the zinc-finger class of DNA-binding transcription factors [14]. KLFs contain three conserved zinc-fingers within the carboxy-terminus which bind a consensus 5′-C(A/T)CCC-3′ motif in the promoters and enhancers of various genes [15]. The amino-terminus is involved in transcriptional activation and repression as well as protein-protein interaction [15, 16]. To date, 18 members have been identified, and our initial insights linking the KLF gene family to metabolism were gleaned from studies implicating KLF15 as a regulator of adipogenesis [17, 18]. More recently, we provided the inaugural evidence implicating KLF15 as a core component of the transcriptional circuitry that governs cardiac metabolism [19]. In particular, KLF15-null hearts are characterized by a significant reduction in FAO with a concomitant increase in glucose oxidation [19]. Unbiased transcriptional profiling revealed a KLF15-depedent signature for myocardial substrate metabolism, in particular genes involving lipid flux [19]. These studies coupled with the work of others have led to increasing appreciation that KLFs are, together with NRs, nodal determinants of metabolism.

Given our observation that the KLF15-null heart phenocopies, at both the functional and transcriptional level, the known roles previously ascribed to PPAR*α* in regulating cardiac metabolism, we postulated whether KLF15 and PPAR*α* operate in a coordinated fashion to regulate lipid gene expression. Here, we demonstrate that KLF15 binds to, cooperates with, and is requisite for the ability of PPAR*α* to induce a subset of target genes critical for cardiac lipid oxidation.

## 2. Materials and Methods

### 2.1. Animal Models

Studies dealing with animal use were approved by the Institutional Animal Care and Use Committee at Case Western Reserve University and conducted in strict accordance with the NIH Guide for the Care and Use of Laboratory Animals. Mice were housed in a temperature and humidity controlled barrier facility with a 12-hour light/dark cycle and ad libitum access to water and standard laboratory rodent chow. Transverse aortic constriction (TAC) studies were performed with age- and sex-matched controls (10–14-week-old, male, pure C57Bl/6 background) as previously described [20, 21].

### 2.2. Plasmids and Adenoviruses

An expression plasmid containing the mouse KLF15 cDNA has been described previously [21]. An expression plasmid containing the rat PPAR*α* cDNA was purchased from OriGene. The −1.2 kB promoter region of mouse* Slc27a1* (*Fatp1*) was described previously [19]. The –1.6 kB* Pdk4* promoter-luciferase construct was a gift from Kelly [22]. The generation of an shRNA, against mouse/rat* Klf15*, has been described previously [19]. Amplification and purification of the adenoviral vectors was performed by Welgen, Inc.

### 2.3. Cell Culture

Neonatal rat ventricular myocytes (NRVMs) were isolated from 2-day-old rat pups and maintained under standard conditions as previously described [19, 21, 23, 24]. Isolated NRVMs were cultured for 48 h under quiescent conditions by the inclusion of serum-free DMEM supplemented with 0.1% BSA, 1X ITS, and 1% Pen/Strep prior to experiments. Following quiescence, NRVMs were infected with adenoviral vectors (sh-control or sh-*Klf15*) for 24 hrs. Following transduction, NRVMs were treated with the exogenous addition of either DMSO (vehicle) or 10 *μ*M WY-14643 for additional 24 hrs. Cells were then harvested, RNA was isolated, and QPCR was performed as described below. NIH-3T3 cells were purchased from ATCC (Manassas, VA) and grown in DMEM supplemented with 10% FBS and 1% Pen/Strep.

### 2.4. RNA Extraction and QPCR

Heart tissue samples were disrupted/homogenized in PureZOL (Biorad) in a Tissue-lyser (Qiagen) using stainless steel beads (30 Hz for a total of 4 min). Total RNA was isolated using the Aurum (Biorad) RNA isolation kit according to manufacturer's directions. For cellular samples, total RNA from NRVM was isolated using the high pure RNA isolation kit (Roche) according to manufacturer's directions. For QPCR, total RNA was deoxyribonuclease-treated on-column and transcribed to complementary DNA using iScript (Biorad) following manufacturer's protocol. QPCR was performed with the TaqMan method (using the Roche Universal Probe Library System) on an ABI Step One Plus Real-Time PCR System. Relative expression was calculated using the ΔΔ*Ct* method with normalization to constitutive genes as indicated in each figure. Specific primer/probe sequences are available on request.

### 2.5. Coimmunoprecipitation

CoIP was performed from nuclear protein extracts. Nuclear protein from NIH-3T3 cells was prepared using the Ne-PER kit (Pierce) according to manufacturer's instructions. For each IP, 500–7500 *μ*g nuclear protein was loaded in IP dilution buffer (25 mM Tris-HCl, pH 7.4, 137 mM NaCl, 0.5% NP40, 0.5 mM EDTA supplemented with protease inhibitors) and immunoprecipitated at 4C overnight with EZview Red Anti-FLAG M2 Affinity Gel (Sigma) according to manufactures instructions. Immune complexes were washed extensively and eluted first with 3X Flag peptide (Sigma) then in SDS sample buffer and boiled. Immunoprecipitated and input proteins were run on SDS-PAGE and immunoblotted with the following antibodies as indicated: goat polyclonal anti-KLF15 (Abcam) and anti-Flag (Sigma, Clone M2). Secondary HRP-conjugated antibodies and ECL-plus chemiluminescent detection reagent were from Amersham. Immunoblots presented are from a representative experiment that has been repeated three times.

### 2.6. Promoter-Luciferase Studies

Transient transfections of indicated promoter and expression plasmids using cultured NIH-3T3 cells at 70% confluence were carried out using Fugene6 (Roche and Promega) following manufacturer's instructions and bioluminescence recorded on a Veritas luminometer (Turner Biosystems) as previously described [19, 21, 25].

### 2.7. Cellular Lipid Oxidation

Rates of oxygen consumption from exogenous lipid oxidation in NRVM were measured using a Seahorse Bioscience XFp Extracellular Flux Analyzer. Following isolation described above, NRVMs were seeded at a cellular density of 80,000 cells/well and cultured for 48 h under quiescent conditions by the inclusion of serum-free DMEM supplemented with 0.1% BSA, 1X ITS, and 1% Pen/Strep. Following quiescence, NRVMs were infected with adenoviral vectors (sh-control or sh-*Klf15*) for 24 hrs. Following transduction, NRVM media was replaced with substrate limited media according to manufacturer's directions and treated with the exogenous addition of either DMSO (vehicle) or 10 *μ*M WY-14,643 for an additional 24 hrs. One hour prior to measuring oxygen consumption, NRVM substrate limited media was replaced with XF assay medium and maintained in a non-CO_2_ incubator for 1 hr followed by the acute addition of exogenous BSA-conjugated palmitate (150 nM, Seahorse Bioscience). NRVMs with sensor cartridges were placed in the XFp Analyzer and manufacturer's directions for the Mito Stress Test kit was followed. In brief, mitochondrial function was determined by the sequential injection of oligomycin A (2.5 *μ*g/mL), FCCP (1 *μ*M), and antimycin A (4 *μ*M) in combination with rotenone (2 *μ*M). Following each experiment, total cellular protein was determined using the Pierce BCA Protein Assay.

### 2.8. Data Analysis

All results are expressed as means, and error bars depict SEM. For experiments comparing the means of two normally distributed groups, two-tailed Student's* t*-test for unpaired data was used. For experiments comparing the means of normally distributed groups with multiple treatments, two-way analysis of variance (ANOVA) with the Bonferroni post hoc test was used. Statistical significance was defined as *P* < 0.05.

## 3. Results

### 3.1. KLF15 and PPAR*α* Expression Are Regulated by Common Physiological and Pathological Stimuli

**Figure 1:**
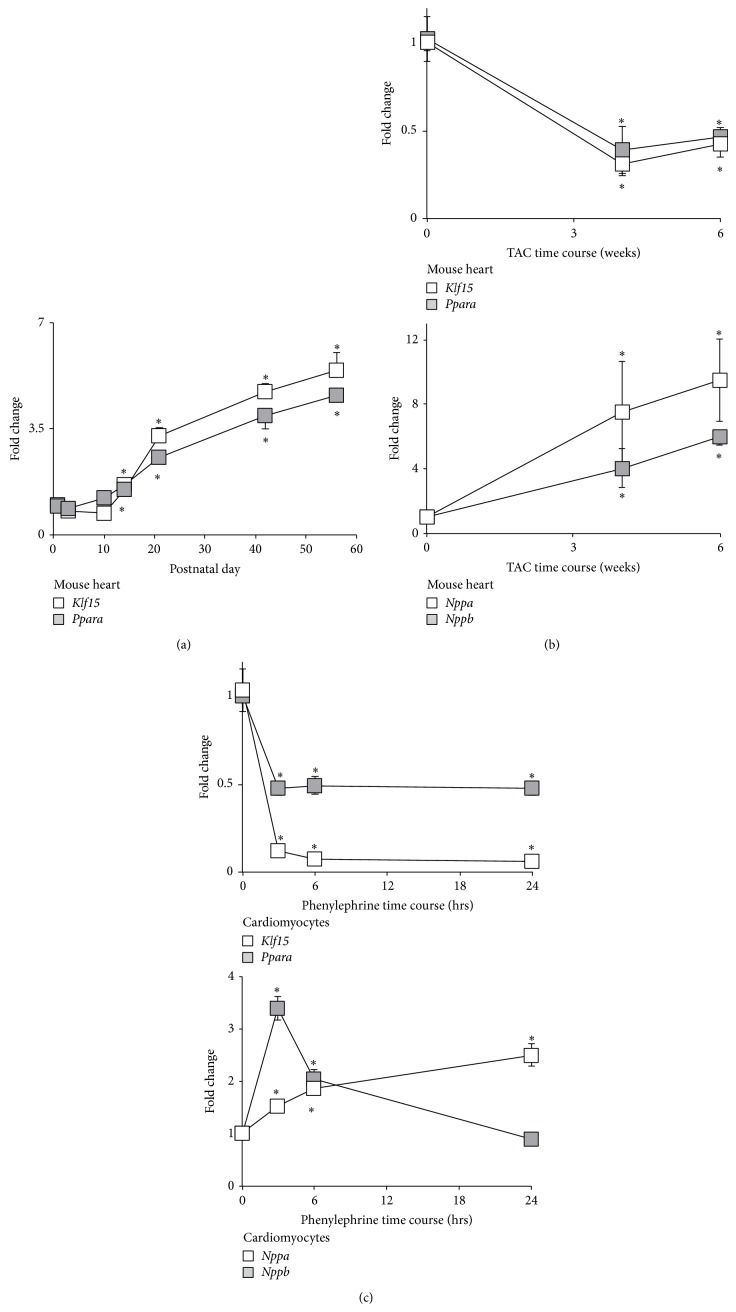
Expression of KLF15 and PPAR*α* are regulated by physiological and pathological stimuli. (a)* Klf15* and* Ppara* expression in mouse heart during postnatal maturation (*n* = 4). ^*^ *P* < 0.05 versus day 1. (b)* Klf15* and* Ppara* (top panel) and Nppa/ANF and Nppb/BNP (bottom panel) expression in murine heart tissue in sham (*t* = 0 weeks) versus transaortic constriction (TAC) at multiple time points (*n* = 3-4). ^*^ *P* < 0.05 versus sham. (c)* Klf15* and* Ppara* (top panel) and Nppa/ANF and Nppb/BNP (bottom panel) expression in neonatal rat ventricular cardiomyocytes following exogenous addition of vehicle (*t* = 0 hours) versus phenylephrine (100 *μ*M) at multiple time points (*n* = 6). ^*^ *P* < 0.05 versus vehicle. Values normalized to* cyclophilin-B *(*Ppib*).

To glean insights into the potential of KLF15 and PPAR*α* operating in a coordinated fashion to regulate lipid gene expression, we first assessed their mRNA expression in conditions where lipid utilization is augmented (i.e., postnatal maturation) or reduced (response to hypertrophy) [1, 2, 26, 27]. As shown in Figure 1, both* Klf15* and* Ppara* levels were increased after birth in murine rodent hearts (Figure 1(a)). By contrast, heart* Klf15* and* Ppara* levels were reduced following 4 and 6 weeks of transaortic constriction, a hypertrophic state characterized by reduced lipid utilization (Figure 1(b), top panel). Additionally, this pattern of gene expression was recapitulated at the cellular level using cultured neonatal rat ventricular cardiomyocytes (NRVM) in the presence of the well-established prohypertrophic stimuli phenylephrine, thus demonstrating a cell autonomous effect (Figure 1(c), top panel). Importantly, these alterations in* Klf15* and* Ppara* levels under pathological conditions paralleled the gene expression of known hypertrophic markers including* Nppa*/ANF and* Nppb*/BNP (Figures 1(b) and 1(c), bottom panels). Collectively, these data suggest that cardiac KLF15 and PPAR*α* levels are regulated by physiologic/pathologic stimuli and that their expression correlates with states of enhanced cardiac lipid utilization.

### 3.2. KLF15 Interacts and Cooperates with PPAR*α*

**Figure 2:**
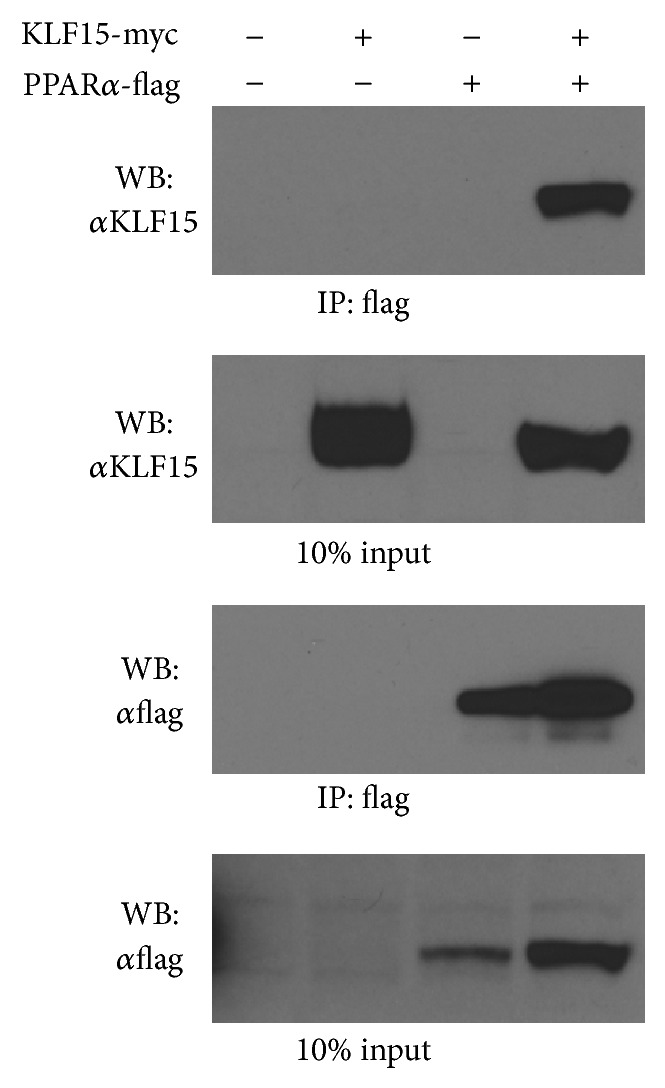
KLF15 and PPAR*α* direct interaction. Co-IP demonstrating protein-protein interaction between overexpressed KLF15 (Myc-tagged) and PPAR*α* (Flag-tagged) in nuclear extracts of NIH-3T3 cells. Immunoblots presented are from a representative experiment that has been repeated three times.

**Figure 3:**
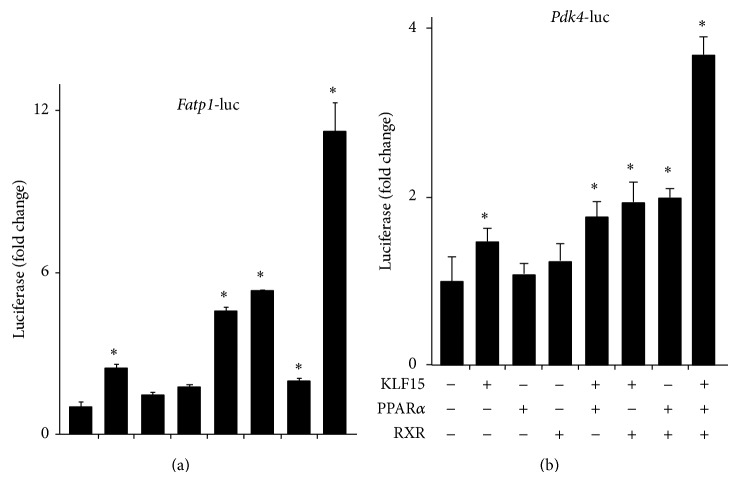
KLF15 cooperates with PPAR*α* to regulate canonical target promoters. Cooperative promoter induction between KLF15 and PPAR*α* on −1.2 kb Fatp1-luciferase (a) and −1.6 kb* Pdk4*-luciferase (b) in transfected NIH-3T3 cells (*n* = 6). ^*^ *P* < 0.05 versus mock transfection.

Current and previous observations suggest that the cardiac expression pattern of KLF15, its gene targets, and its metabolic effects paralleled those previously ascribed to PPAR*α* [13, 19]. A potential mechanism by which this occurs is KLF15 that acts to transcriptionally regulate PPAR*α* expression. However, KLF15 minimally affects PPAR*α* expression* in vivo* [19]. As such, these data suggest that KLF15 and PPAR*α* may, instead, cooperate on common targets to regulate cardiac lipid flux. As a first step to elucidate this possibility, we performed coimmunoprecipitation studies. As shown from the representative experiment in Figure 2, direct physical interaction between epitope-tagged KLF15 and PPAR*α* is confirmed when coexpressed in NIH-3T3 cells. Next, cotransfection studies were undertaken to test cooperativity directly. As shown in Figure 3, KLF15 and PPAR*α* cooperate in a synergistic manner to induce the* Fatp-1* (Figure 3(a)) and* Pdk4* (Figure 3(b)) promoter. Collectively, these data support the hypothesis that a KLF15-PPAR*α* molecular axis coordinates the expression of genes important for myocardial lipid utilization.

### 3.3. Ligand Induced PPAR*α* Gene Targets and Lipid Oxidation Are KLF15 Dependent

**Figure 4:**
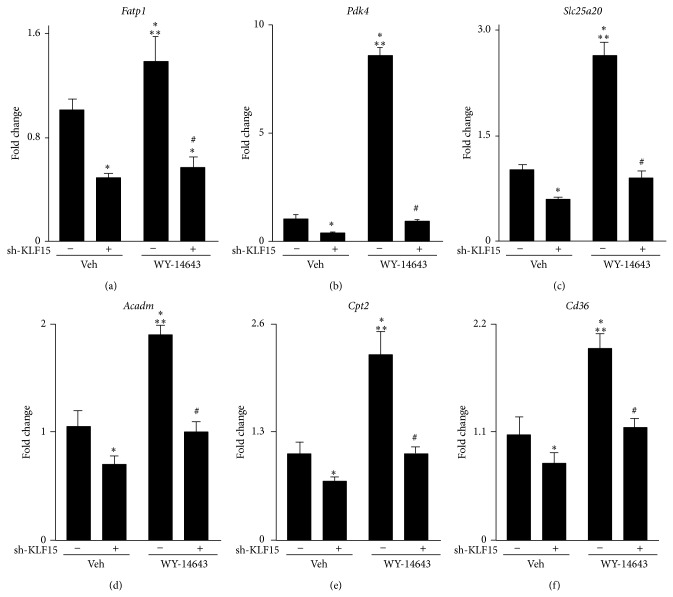
Ligand induced PPAR*α* gene targets are KLF15 dependent.* Fatp1* (a),* Pdk4* (b),* Slc25a20* (c),* Acadm* (d),* Cpt2* (e), and* Cd36* (f) expression (qPCR) in neonatal rat ventricular cardiomyocytes after sh-RNA mediated* Klf15* silencing in the absence or presence of the PPAR*α*-specific ligand WY-14,643 (10 *μ*M, 24 hrs). (*n* = 6). ^*^ *P* < 0.05 versus vehicle. ^**^ *P* < 0.05 versus sh-KLF15. ^#^ *P* < 0.05 versus WY-14643. Values normalized to* Ppib*.

To determine the dependency of KLF15 in regulating canonical PPAR*α* transcriptional targets, cultured NRVMs sufficient or deficient in KLF15 were treated with the PPAR*α*-specific agonist WY-14,643. As expected, WY-14,643 treatment induced canonical PPAR*α* targets such as* Fatp1*,* Pdk4*,* Slc25a20*,* Acadm*,* Cpt2*, and* Cd36* (Figures 4(a)–4(f)). Strikingly, however, the PPAR*α*-agonist response was markedly blunted with* Klf15* silencing (>96% reduction in* Klf15* (data not shown) (Figures 4(a)-4(f)). Importantly, acute silencing of* Klf15* had no effect on endogenous* Ppara* expression (data not shown). Moreover, the use of synthetic agonists in these set of experiments strongly suggest that the reduced PPAR*α* transcriptional activity phenotype observed in the absence of KLF15 is not due to loss of an endogenous PPAR ligand but likely due to cooperative interactions between these two factors.

**Figure 5:**
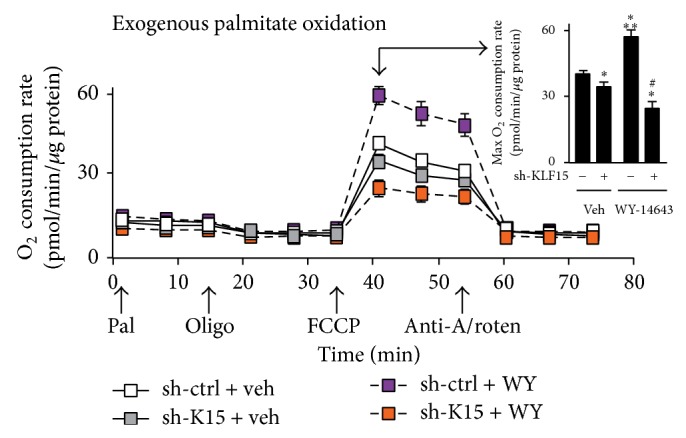
Ligand induced PPAR*α* lipid oxidation is KLF15 dependent. Oxygen consumption rate (pmol/min) in neonatal rat ventricular cardiomyocytes after sh-RNA mediated* Klf15* silencing in the absence or presence of the PPAR*α*-specific ligand WY-14,643 (10 *μ*M, 24 hrs). Arrows along *x*-axis indicate acute addition of BSA-conjugated palmitate (Pal, 150 nM), ATP synthase inhibitor oligomycin (Oligo, 2.5 *μ*g/mL), uncoupler FCCP (1.5 *μ*M), complex III inhibitor antimycin A (4 *μ*M)/complex I inhibitor rotenone (2 *μ*M) (Anti-A/Roten).* Inset*: maximal oxygen consumption rate at* t* = 40 min following addition of uncoupler FCCP (*n* = 6–9). ^*^ *P* < 0.05 versus vehicle. ^**^ *P* < 0.05 versus sh-KLF15. ^#^ *P* < 0.05 versus WY-14643. Values normalized to total cellular protein.

As the above results establish the importance of KLF15 in mediating ligand-induced PPAR*α* dependent gene expression, we next sought to determine the KLF15 dependency in regulating PPAR*α* mediated cellular lipid oxidation. Cultured NRVMs sufficient or deficient in KLF15 were treated with the PPAR*α*-specific agonist WY-14,643 followed by the acute addition of BSA-conjugated palmitate. As expected, WY-14,643 treatment significantly induced the NRVM maximal oxygen consumption rate in the presence of exogenously added palmitate (Figure 5). While* Klf15* silencing produced a slight, yet significant, decrease in the maximal oxygen consumption rate, quite strikingly, however, the PPAR*α*-agonist response was markedly blunted in the absence of* Klf15* (Figure 5). Taken together, these results support the notion that KLF15 and PPAR*α* cooperate to regulate gene expression critical for lipid oxidation.

## 4. Discussion

The heart's metabolic adaptation to nutrient availability is requisite for survival [1, 2]. This adaptation is coordinated, at least in part, at the gene regulatory level, and the current study establishes a novel molecular module involving the coordinated action of KLF15 and PPAR*α* that governs metabolic gene expression in the heart. Using expression profiling, we observe that cardiac KLF15 and PPAR*α* expression are increased during states of heightened demand for lipid oxidation (i.e., postnatal maturation). In contrast, during states of decreased reliance on lipid oxidation, both KLF15 and PPAR*α* expressions are reduced. Additionally, KLF15 binds to and synergizes with PPAR*α* to induce a subset of genes critical to cardiac energetics. Finally, we provide evidence to support the fact that KLF15 is required for the PPAR*α* mediated gene induction as well as lipid oxidation in isolated cardiomyocytes. When considered alongside previous observations, the current work implicates a novel transcriptional circuitry involving KLF15 and PPAR*α* that coordinates metabolic gene expression and lipid utilization in the heart and thus adds to a growing body of evidence suggesting that molecular control of metabolism governs heart function in health and disease.

KLF15 is highly expressed in metabolically active tissues (e.g., heart, skeletal muscle, and liver), and previous work from our group has shown that cardiac KLF15 expression is induced with fasting and significantly attenuated in rodent and human samples of heart failure [14, 19, 28, 29]. Systemic deletion of KLF15 results in reduced fatty acid oxidation in the isolated working heart model as well as enhanced heart failure following pressure overload [19, 21]. Like KLF15, PPAR*α* expression is robust in tissues dependent on oxidative metabolism and loss-of-function studies reveal a critical role for PPAR*α* in cardiac FAO as well as the hearts response to hypertrophy [8, 13, 30]. Moreover, cardiac specific PPAR*α*-transgenic mice display enhanced FAO, increased lipid droplets, and ventricular hypertrophy [11]. As such, the cardiac expression patterns of both KLF15 and PPAR*α*, and their effects on metabolism and gene expression, mirror one another suggesting that both transcription factors act in synchrony to regulate cardiac metabolism. Given that loss of KLF15 minimally affects PPAR*α* expression, and vice versa (data not shown), this suggests these transcription factors act in a cooperative fashion to exert their effects. This notion is supported by evidence presented in Figures 4 and 5. Additionally, it is important to note that while KLF15 deficient hearts demonstrate a paucity of lipid droplets in the heart [19], suggesting reduced ligand for optimal PPAR*α* activity, the results presented in Figure 4 circumvent this possibility with the use of a synthetic ligand specific for PPAR*α*, thus providing additional evidence supporting a cooperative mechanism of action.

Moreover, several studies provide compelling evidence supporting this molecular mechanism* in vivo* as well as its importance in cardiac function/metabolism under physiologic or pathologic conditions. While pathways regulating FA utilization are essential for cardiac homeostasis, they can be usurped in states of nutrient excess to confer maladaptive effects. For example, in states such as diabetes, inadequate insulin signaling is interpreted by tissues as a state of “starvation” despite the availability of excess nutrients [7]. The denouement of this disturbed metabolic milieu is the development of a diabetic cardiomyopathy, which is characterized by excess fatty acid uptake/lipotoxicity, mitochondrial dysfunction, and ROS production culminating in structural and functional derangements [31]. Indeed, cardiac overexpression of PPAR*α* confers a phenotype that has many of the features of a diabetic cardiomyopathy [11, 32]. Conversely, PPAR*α* deficiency is protective in models of diabetic cardiomyopathy [32]. Moreover, KLF15 knockout mice are protected against insulin resistance under high-fat feeding conditions [33]. Given these data, it is reasonable to suggest that the KLF15-PPAR*α* molecular module described in the current study is a critical determinant of a myopathic phenotype* in vivo*, and future studies aimed at elucidating the interdependency between KLF15 and PPAR*α*, through the generation of compound mutant models, will therefore prove beneficial.

As stated above, we show that knockdown of KLF15 attenuates the ability of a synthetic PPAR*α* ligand to maximally induce target gene expression (Figure 4). Importantly, however, this agonist-mediated effect is blunted, not absent, with KLF15 silencing. While significant, many of the PPAR*α* induced target genes demonstrate ~50% reduction in the absence of KLF15 suggesting that KLF15 and PPAR*α* are part of a hierarchical transcriptional complex involving additional DNA-binding transcription factors or coactivators/repressors. A well-established coactivator of PPAR*α* is peroxisome proliferator-activated receptor gamma coactivator 1 (PGC1) [34]. Moreover, we have previously demonstrated that KLF15 interacts and synergizes with the chromatin remodeling acetyltransferase p300 to regulate transcriptional targets including* Fatp1* and* Pdk4* [19]. Additionally, previous reports have demonstrated that PPAR*α* interacts with p300 [35]. As loss of cardiac KLF15 or PPAR*α* expression is associated with reduced lipid metabolism and contractile dysfunction [13, 19, 21, 36], when taken in the context of our current findings, it is likely that reduced expression of either KLF15 or PPAR*α*, or both, liberates p300 and thus promotes a closed chromatin configuration on gene targets involved in the lipid oxidation pathway. Supporting this model are recent observations that suggest that p300 is a positive regulator of cardiac hypertrophy, a pathological state characterized by reduced lipid flux [37, 38]. It is therefore reasonable to suggest that KLF15 and PPAR*α* are part of a much larger transcriptional complex involving the dynamic interplay between coactivators/repressors along with chromatin remodeling proteins that mediate genetic regulation of metabolic transcriptional targets in response to environmental cues.

Additionally, our results presented in Figure 3 are of particular interest given the known role of RXR as an obligate heterodimeric partner for nuclear receptors, in general, and PPAR*α*, in particular [8]. Our results suggest that KLF15/RXR is at least as efficient in mediating promoter induction of target genes compared to KLF15/PPAR*α* or PPAR*α*/RXR, again suggesting the possibility that KLF15 is part of a broader transcriptional complex with other DNA-binding partners (i.e., PPAR*α* and potentially RXR), as well as coactivators mentioned above, to elicit appropriate gene responses. RXR expression is robust in metabolically active tissues, and loss of function studies highlight an important developmental role as RXR*α*-KO mice die between E13.5 and E16.5 due to cardiac ventricle hypoplasia, wall thinning, and defects in ventricular septation [39–41]. Mechanistically, RXR functions in multiple ways: as a heterodimer required for DNA binding but not acting as a receptor-ligand (termed nonpermissive heterodimers); as a functional component of the heterodimer mediated by rexinoid activation of RXR (termed permissive heterodimers); and as a functional homodimer coupled to rexinoid signaling and independent of other nuclear receptors [39, 41]. The latter mechanism of action is highlighted by a recent report that demonstrates that RXR-dependent induction of* Pdk4* is independent of PPAR*α* [42]. As such, when taken together with our current observations, future experiments should focus on the role of the KLF15/PPAR*α* complex in mediating RXR dependent and independent transcriptional gene programs.

Finally, this work adds to the growing appreciation that KLFs and NRs are pervasive, operating in a coordinated fashion to regulate tissue and organismal physiology [43]. While previous reports implicate a molecular mechanism involving KLFs and NRs in the context of, but not limited to, neuronal differentiation and ischemic stroke [44, 45], smooth muscle biology [46], prenatal lung differentiation [47, 48], hepatic steatosis [49], endometriosis [50], and browning of adipocytes [51], this report is the first demonstration that KLFs and NRs, in general, and KLF15 and PPAR*α*, in particular, coordinate metabolic gene expression and function in the cardiomyocyte. Moreover, recent observations demonstrate that KLF15 is a glucocorticoid receptor (GR) target and acts as a feed-forward enhancer or inhibitor of GR signaling in the lung and skeletal muscle [52, 53]. Also, the Nagai laboratory demonstrated KLF5 as a critical regulator of skeletal muscle lipid metabolism and systemic energy homeostasis through a molecular mechanism involving PPAR*δ* [54]. Specially, ligand induction of PPAR*δ* recruits the SUMO-protease SENP1 which deSUMOylates KLF5 and relieves tonic transcriptional repression and thus synergizes with PPAR*δ* to promote transactivation of promoter elements. Taken together, the existence of a KLF15-PPAR*α* molecular module elucidated here, when considered alongside previous observations, adds to a burgeoning body of evidence that suggest that KLFs and NRs display a dynamic molecular interplay to regulate cellular physiology.

## 5. Conclusions

In conclusion, the present study demonstrates that KLF15 and PPAR*α* expression correlates with states of enhanced cardiac lipid utilization. Moreover, these transcription factors form a protein-protein interaction and cooperate on target promoters. Finally, the PPAR*α* mediated induction of canonical gene targets and lipid oxidation is blunted in the absence of KLF15. These observations thus support a novel molecular mechanism that governs cardiac fuel utilization that can be exploited for future studies and potential therapeutic gain.
